# Impact of PEGylated Liposomal Doxorubicin and Carboplatin Combination on Glioblastoma

**DOI:** 10.3390/pharmaceutics14102183

**Published:** 2022-10-13

**Authors:** Mohsen Ghaferi, Aun Raza, Maedeh Koohi, Warda Zahra, Azim Akbarzadeh, Hasan Ebrahimi Shahmabadi, Seyed Ebrahim Alavi

**Affiliations:** 1 Immunology of Infectious Diseases Research Center, Research Institute of Basic Medical Sciences, Rafsanjan University of Medical Sciences, Rafsanjan 7717933777, Iran; 2 School of Pharmacy, Jiangsu University, Zhenjiang 212013, China; 3 Department of Surgery, Nishtar Medical University and Hospital, Multan 60000, Pakistan; 4 Department of Pilot Nanobiotechnology, Pasteur Institute of Iran, Tehran 1316943551, Iran

**Keywords:** glioblastoma, PEGylated liposome, nanoparticles, nanotechnology

## Abstract

Glioblastoma is an incurable cancer with a 5-year survival chance of less than 5%. Chemotherapy is a therapeutic approach to treating the disease; however, due to the presence of the blood–brain barrier (BBB), the probability of success is low. To overcome this issue, nanoparticles are promising carriers for crossing the BBB and delivering drugs to the tumor. In this study, the anticancer efficacy of doxorubicin (DOX) and carboplatin (CB) loaded into polyethylene glycol (PEG)ylated liposome nanoparticles (PEG-Lip) and in treating brain cancer was evaluated in vitro and in vivo. The results demonstrated that PEG-Lip-DOX/CB with a size of 212 ± 10 nm was synthesized that could release the loaded drugs in a controlled manner, from which 56.3% of the loaded drugs were released after 52 h. In addition, PEG-Lip-DOX/CB could significantly increase the cytotoxicity effects of the drugs against rat glioma C6 cells (IC_50_: 8.7 and 12.9 µM for the drugs-loaded nanoparticles and DOX + CB, respectively). The in vivo results also demonstrated that PEGylated liposomes, compared to non-PEGylated liposomes (Lip) and DOX + CB, were more efficient in increasing the therapeutic effects and decreasing the side effects of the drugs, in which the survival times of the glioblastoma-bearing rats were 39, 35, and 30 days in the PEG-Lip-DOX/CB, Lip-DOX/CB, and DOX + CB receiver groups, respectively. In addition, the weight loss was found to be 8.7, 10.5, and 13%, respectively, in the groups. The results of the toxicity evaluation were also confirmed by histopathological studies. Overall, the results of this study demonstrated that the encapsulation of DOX and CB into PEG-Lip is a promising approach to improving the properties of DOX and CB in terms of their therapeutic effects and drug side effects for the treatment of glioblastoma.

## 1. Introduction

Glioblastoma is one of the most malignant tumors affecting the brain and constitutes more than 60% of all brain tumors in adults [1]. Glioblastoma, with a global incidence of 10:100,000, is responsible for 2.5% of deaths owing to all cancers [2]. Ionizing radiation is the strongest risk factor for this disease [3]. Despite progress in different therapies for the treatment of this disease, glioblastoma is still an incurable disease with a poor prognosis. On average, patients die 14 months after diagnosis, and the 5-year survival chance is less than 5%. For this reason, the development of new therapies to improve the prognosis of the disease is urgently needed [1]. The primary treatment is usually surgery and tumor removal, followed by chemotherapy and radiotherapy [1]. However, the blood–brain barrier (BBB) is an obstacle in reaching the chemotherapeutics for brain tumors [4]. In addition, short plasma half-life, enzymatic degradation, the development of innate or other immune responses, the insufficient concentration of drugs in or around the tumors, and trouble in targeting and attaining the controlled release of drugs in the tumors are further challenges to the treatment of brain tumors through the intravenous administration of drugs [5]. These problems cause systemic side effects and difficulty in preparing appropriate doses for the efficient treatment of brain tumors [5]. In this regard, the application of nanoparticles has been considered as an appropriate tool to improve the efficiency of drug delivery, increase the therapeutic effects, and decrease the drugs’ side effects [5,6,7,8,9]. The shape, size, and surface of nanoparticles could be modified to overcome the problems of drug delivery to the brain. Nanoparticles can be modified with suitable ligands and molecules to improve their efficiency for targeted treatment. Their features can be optimized for brain drug delivery purposes through intravenous, intranasal, or local routes. For intravenous delivery, the surface of nanoparticles or their features can be modified to increase the blood half-life or BBB penetration of cargos [5]. Liposomes are one of the nanoparticles used for drug delivery.

Liposomes are lipid-based nanoparticles with an aqueous core. They can be used for the delivery of hydrophilic and hydrophobic drugs [10]. The short serum half-life of liposomes limits their application, which can be resolved through conjugating them to polyethylene glycol (PEG). PEG is a Food-and-Drug-Administration-approved polymer for human use that can improve the aqueous solubility of conjugates [11,12,13]. The PEGylation of liposomes could increase the blood circulation time of liposomes by providing steric stability, ensuring their higher tumor accumulation [14,15]. Doxorubicin (DOX) and carboplatin (CB) are two anticancer drugs used for the treatment of glioblastoma [4]. However, the clinical application of these drugs is associated with various adverse effects such as cardiotoxicity, hepatotoxicity, myelosuppression [16], high systemic toxicity, including nephrotoxicity, central neurotoxicity, and peripheral neuropathy [17].

This study aimed to develop a stable nanoplatform for the co-delivery of DOX and CB with decreased drug toxicity and improved anticancer effects against glioblastoma cells in vitro and in vivo using PEGylated liposome nanoparticles. As far as we know, the development of a nanosystem for the co-delivery of DOX and CB has not been reported until now; however, investigating the therapeutic application of PEGylated liposomal DOX in combination with the standard CB has been reported for the treatment of ovarian cancer [18]. Pujade-Lauraine et al. [18] evaluated the efficacy and safety of PEGylated liposomal DOX with standard CB (CD arm), compared with standard CB and paclitaxel (CP; CP arm), in the treatment of patients with platinum-sensitive relapsed/recurrent ovarian cancer. The results demonstrated that the CD arm, compared to the CP arm, was more efficient in improving progression-free survival (PFS) by 16.8%. In addition, the results of the toxicity evaluation demonstrated that in the CP arm, compared to the CD arm, more alopecia (83.6% vs. 7%), hypersensitivity reactions (18.8% vs. 5.6%), and sensory neuropathy (26.9% vs. 4.9%), were observed, while hand-foot syndrome (grade 2 to 3, 12.0% vs. 2.2%), nausea (35.2% vs. 24.2%), and mucositis (grade 2–3, 13.9% vs. 7%) was observed more in the CD arm, compared to that of the CP arm. Overall, the results of this study demonstrated the superiority of the CD arm, compared to the CP arm, in terms of improving the PFS and therapeutic index. In the current study, DOX- and CB-loaded nanoparticles were synthesized (liposomal DOX: Lip-DOX, PEGylated liposomal DOX: PEG-Lip-DOX, liposomal CB: Lip-CB, PEGylated liposomal CB: PEG-Lip-CB, liposomal DOX and CB: Lip-DOX/CB, PEGylated liposomal DOX and CB: PEG-Lip-DOX/CB) using the reverse-phase evaporation method and characterized in terms of size, morphology, drug loading efficiency, and drug release using Zetasizer, scanning electron microscopy (SEM), and spectrophotometer instruments. Next, the biological effects of the formulations were evaluated in vitro using 3-(4,5-dimethylthiazol-2-yl)-2,5-diphenyl-2H-tetrazolium bromide (MTT), cell proliferation, and reactive oxygen species (ROS) assays. The efficacy of the formulation was then evaluated in vivo in the treatment of glioblastoma-bearing rats.

## 2. Materials and Methods

### 2.1. Materials

CB, DOX, egg lecithin, cholesterol, dimethylsulfoxide (DMSO), ethanol (EtOH), sucrose, dialysis bag (cut-off 10 kDa), phosphate buffer saline (PBS), sodium dodecyl sulfate (SDS), hematoxylin and eosin, and 2′-7′ dichlorofluorescin diacetate (DCFH-DA) were purchased from Sigma-Aldrich (St. Louis, MO, USA). 1,2-distearoyl-sn-glycero-3-phosphoethanolamine-N-[amino(polyethylene glycol)-2000] (DSPE-PEG2000) was purchased from Ponsure Biotechnology (Shanghai, China). Roswell Park Memorial Institute (RPMI)-1640 medium, penicillin and streptomycin antibiotics, and fetal bovine serum (FBS) were purchased from Gibco (Waltham, MA, USA). Rat glioma C6 cells and male albino Wistar rats (200–250 g, 8 weeks old) were supplied by the Pasteur Institute of Iran, Tehran. Deionized water was used throughout the study.

### 2.2. Nanoparticle Preparation

PEG-Lip-DOX, PEG-Lip-CB, and PEG-Lip-DOX/CB nanoparticles were synthesized using the reverse-phase evaporation method [19]. Different concentrations and ratios of lecithin, cholesterol, DOX/CB, and DSPE-PEG2000 (Tables S1–S3) were used to optimize the formulation composition. The best concentrations and ratios were chosen for subsequent studies based on the results of size, polydispersity index (PDI), and drug encapsulation efficiency (EE%) (Table 1). For this purpose, 7.3 mg of DSPE-PEG2000, 21.6 mg of lecithin, 8 mg of cholesterol, and 12 mg of DOX were dissolved in 10 mL of chloroform. The solvent was then removed using a rotary evaporator (Heidolph Co., Schwabach, Germany), and the thin film prepared on the bottom of the flask was suspended into 7.2 mL of PBS (pH 7.4) using a stirrer (180 RPM, 10 min). Next, to reduce the size and to produce unilamellar vesicles, the prepared vesicles were sonicated in a bath sonicator (Bandelin Sonorex Digitec, Berlin, Germany; 60 Hz) for 30 min. PEG-Lip-CB was synthesized with the same method, except that 12 mg of CB was used instead of DOX. In addition, PEG-Lip-DOX/CB was synthesized with this method, except that 6 mg of each drug (DOX and CB) was used. Moreover, Lip-DOX, Lip-CB, and Lip-DOX/CB were synthesized with the same method without adding the DSPE-PEG2000 into the reaction medium. The blank nanoparticles (liposome: Lip and PEGylated liposome: PEG-Lip) were synthesized with the same method, except that the drugs were not added to the reaction medium (Table 1). 

### 2.3. Nanoparticle Characterization

#### 2.3.1. Dynamic Light Scattering Analysis

The size, PDI, and zeta potential of Lip, Lip-DOX, Lip-CB, Lip-DOX/CB, PEG-Lip, PEG-Lip-DOX, PEG-Lip-CB, and PEG-Lip-DOX/CB were measured and compared using the DLS method and a Zetasizer instrument (ZEN 3600, Malvern Instruments Ltd., Worcestershire, UK). For this purpose, a suspension (0.5 mg/mL in PBS) of the nanoparticles was separately prepared and introduced to the Zetasizer.

#### 2.3.2. Scanning Electron Microscopy Analysis

To evaluate and compare the morphology of Lip, Lip-DOX, Lip-CB, Lip-DOX/CB, PEG-Lip, PEG-Lip-DOX, PEG-Lip-CB, and PEG-Lip-DOX/CB, an SEM (XL30, Philips, The Netherlands) instrument was used. For this purpose, 200 µL of the suspensions of each nanoparticle were centrifuged (13,000 RPM, 30 min, 4 °C) to obtain the pellets. The pellets were resuspended in 200 µL of a 15 mg/mL sucrose solution, as a cryoprotectant, and lyophilized [20]. The resulting powders were coated with a thin layer of gold and imaged by the SEM instrument.

#### 2.3.3. Loading Capacity and Encapsulation Efficiency

The drug-loading efficiency (LE%) and EE% for Lip-DOX, Lip-CB, Lip-DOX/CB, PEG-Lip-DOX, PEG-Lip-CB, and PEG-Lip-DOX/CB were calculated indirectly [21], in which the drug content in the supernatant was calculated and subsequently subtracted from the initial drug used; thus, the content of the drug loaded into the nanoparticles was obtained [22]. For this purpose, 0.5 mL of each formulation was centrifuged (13,000 RPM, 30 min, 4 °C) to obtain their supernatants. The CB and DOX concentration in the supernatants was measured using spectrophotometry and a standard curve at 235 and 480 nm for CB and DOX, respectively. The standard curve of CB and DOX was plotted spectrophotometrically at 235 and 480 nm, respectively. For this purpose, the drug concentrations of 2, 1, 0.5, 0.25, 0.125, 0.0625, and 0.03125 mg/mL for CB and DOX were prepared and their absorbances were measured. The standard curve (drug concentration vs. optical density (OD)) was then plotted. For this purpose, three stock solutions of DOX and CB with a concentration of 2 mg/mL were prepared in PBS (pH 7.4), and from each stock, drug concentrations of 2, 1, 0.5, 0.25, 0.125, 0.0625, and 0.03125 mg/mL were prepared in PBS (pH 7.4). The absorbances of the solutions were then read at 235 and 480 nm for CB and DOX, respectively, against PBS (pH 7.4) as a blank. The mean values of absorbances for each concentration were calculated, the standard curve as the drug concentration versus optical density (OD) was plotted, and the line equation was obtained. According to the line equation and OD obtained from the supernatant of the drug-loaded nanoparticles, the concentration of the drug in the supernatant was measured. EE% and LE% were then calculated using the following formulate:

(1) $$EE\%=\frac{Initial\;drug\;concentration\;\left(\mathrm{mg}\right)-Drug\;concentration\;in\;supernatant\;\left(\mathrm{mg}\right)}{Initial\;drug\;concentration\;\left(\mathrm{mg}\right)}\times 100$$

(2) $$LE\%=\frac{Loaded\;drug\;in\;nanoparticles\;\left(\mathrm{mg}\right)}{Weight\;of\;nanoparticles\;\left(\mathrm{mg}\right)}\times 100$$

### 2.4. Release Study

The profile of drug release from Lip-DOX, Lip-CB, Lip-DOX/CB, compared to PEG-Lip-CB, PEG-Lip-DOX, and PEG-Lip-DOX/CB, was investigated at pH 7.4 using a dialysis membrane technique. For this purpose, 2 mL of each nanoformulation was centrifuged (13,000 RPM, 30 min, 4 °C) and the pellets were obtained. The pellets (equal to 2.8 and 2.9 mg of DOX and CB in Lip-DOX and Lip-CB, respectively, 1.3 and 1.4 mg of DOX and CB, respectively, in Lip-DOX/CB, 2.9 and 3 mg of DOX and CB in PEG-Lip-DOX and PEG-Lip-CB, respectively, and 1.3 and 1.4 mg of DOX and CB, respectively, in PEG-Lip-DOX/CB) were individually resuspended in 5 mL of PBS and transferred into six individual dialysis bags. In addition, 3 mg of DOX and 3 mg of CB were individually dissolved in 5 mL of PBS and transferred into two dialysis bags. In addition, 1.4 mg of DOX and CB was dissolved in 5 mL of PBS and transferred into a dialysis bag. The bags were separately immersed in the release media (100 mL of PBS) and stirred (150 RPM, room temperature). At the predetermined time intervals (0.25, 0.5, 1, 2, 6, 10, 14, 20, 26, 34, 42, and 52 h), 1 mL of the release media was replaced with 1 mL of fresh PBS. The drug concentrations in the collected samples were measured using spectrophotometry at 235 and 480 nm, and the cumulative curve of drug release versus time was plotted using the formula below:

(3) $$Drug\;release\;\left(\%\right)=\frac{Mass\;of\;the\;released\;drug\;from\;nanoparticles\;\left(\mathrm{mg}\right)}{Mass\;of\;the\;loaded\;drug\;in\;nanoparticles\;\left(\mathrm{mg}\right)}\times 100$$

The drug release kinetics were also determined using various mathematical models, including zero order, first order, Higuchi, and Korsmeyer–Peppas.

### 2.5. Cell Viability Study

The cell viability effects of DOX, CB, DOX + CB, Lip-DOX, Lip-CB, Lip-DOX/CB, PEG-Lip-CB, PEG-Lip-DOX, and PEG-Lip-DOX/CB were measured and compared in vitro using an MTT assay and rat glioma C6 cells. In addition, the cell viability effects of Lip and PEG-Lip at the different concentrations of 10, 20, 40, and 80 mg/mL were evaluated to determine the non-toxic concentrations of the nanoparticles. To evaluate the cell viability effects of the formulations, the cells (10^4^/well) were seeded in 96-well plates containing 100 µL of RPMI-1640 culture medium, supplemented with 10% FBS and 1% penicillin/streptomycin antibiotics (complete media), and incubated (5% CO_2_, 37 °C, for 24 h). The culture media were then replaced with the culture media containing DOX, CB, Lip-DOX, Lip-CB, Lip-DOX/CB, PEG-Lip-CB, PEG-Lip-DOX, and PEG-Lip-DOX/CB at drug concentrations of 2, 4, 8, 16, 32, 64, 128, and 256 µM, and the cells were incubated for 48 h. The culture media were then discarded, and 100 µL of MTT solution (0.5 mg/mL in PBS) was added to each well, and the cells were incubated for 3 h. The MTT solution was next removed, and 100 µL of DMSO was added to each well to dissolve the formazan crystals and incubated for 20 min. The absorbance was then measured at 570 nm using a microplate reader (Biotek, Winooski, VT, USA), and the cell viability (%) was measured using the formula below:

(4) $$Cell\;viability\;\left(\%\right)=\frac{Absorbance_{sample}-Absorbance_{background}}{Absorbance_{negative\;control\;}-Absorbance_{background}}\times 100$$

The negative control and background were the cells incubated with the complete media, and the complete media only, respectively. In addition, the cells treated with SDS (10% *v/v* in water) + 0.1 M HCl were considered as the positive control. In addition, the half-maximal inhibitory concentration (IC_50_) of the formulations was measured using GraphPad Prism Software version 8.00 (GraphPad Software, Inc., San Diego, CA, USA).

### 2.6. Stability Study

The stability of PEG-Lip-CB, PEG-Lip-DOX, and PEG-Lip-DOX/CB, compared to Lip-DOX, Lip-CB and Lip-DOX/CB, was evaluated using the dialysis bag, MTT assay, DLS, and spectrophotometry methods 3 months after their preparation. For this purpose, 5 mL of each formulation equivalent to 36.9 and 29.6 mg of the nanoparticles for the PEGylated and non-PEGylated nanoparticles, respectively, were stored in a 4 °C refrigerator for 3 months, and their profiles of drug release, in vitro antitumor effects, size, PDI, and drug loading efficiency were measured using the methods mentioned above. In addition, the serum stability of PEG-Lip-CB, PEG-Lip-DOX, and PEG-Lip-DOX/CB, compared to Lip-DOX, Lip-CB, and Lip-DOX/CB, was measured in FBS [15]. For this purpose, a suspension of the formulations at a final concentration of 10 mg/mL was prepared in FBS:PBS (45:55% *v/v* ratio) and incubated for 5 h at 37 ℃. The formulation sizes were measured at time intervals of 1, 3, and 5 h.

### 2.7. Reactive Oxygen Species Assay

The efficacy of PEG-Lip-CB, PEG-Lip-DOX, and PEG-Lip-DOX/CB, compared to Lip-DOX, Lip-CB and Lip-DOX/CB, to generate intracellular ROS was investigated using DCFH-DA as a probe. For this purpose, C6 cells (8 × 10^3^ cells/well) were cultured in a 96-well plate containing 100 µL of the complete media to reach 70% confluency. The media was then discarded, and the cells were treated with the complete media containing DOX, CB, DOX + CB, PEG-Lip-CB, PEG-Lip-DOX, PEG-Lip-DOX/CB, Lip-DOX, Lip-CB, and Lip-DOX/CB at an IC_50_ concentration of DOX (16.6 µM), CB (25.4 µM), and DOX + CB (12.9 µM). The cells were incubated (5% CO_2_, 37 °C) for 6 h, the media were removed, and 100 µL of 20 µM DCFH-DA solution was added to the cells. After 30 min incubation in the dark at room temperature, the cells were washed thrice with PBS, and the fluorescence intensity was measured at 485 and 520 nm as the excitation and emission wavelengths, respectively.

### 2.8. Animal Study

The therapeutic effects of PEG-Lip-DOX/CB and Lip-DOX/CB, compared to DOX + CB, on the treatment of glioblastoma-bearing rats were measured by evaluating the survival time, weight changes, serum levels of alanine aminotransferase (ALT), aspartate aminotransferase (AST), BUN, and creatinine. For this purpose, an orthotopic model of a glioblastoma tumor was established in male albino Wistar rats using rat glioma C6 cells according to the previous study [23]. The experiments were approved by the Animal Experimentation Ethical Committee of Rafsanjan University of Medical Sciences, Rafsanjan (Animal Ethical Approval Number: IR.RUMS.REC.1399.279). The animals were kept in a vivarium under controlled conditions of light (12 h light/dark cycle), humidity (50–60%), and temperature (25 ± 2 °C). They were maintained in polypropylene cages and had free access to food and water throughout the experiment. The bottom of the cages was lined with wood husks, which were changed frequently. The animals were allowed to acclimatize to the condition of the vivarium for one week. Two days after tumor cell inoculation, the animals were randomly divided into four groups (*n* = 8) and received PEG-Lip-DOX/CB, Lip-DOX/CB, DOX + CB, and PBS intravenously at a DOX and CB concentration of 1.5 mg/kg at 72 h intervals eight times. The animals in each group were weighed every 3 days and for 40 days. In addition, the animal number in each group was measured until the end of the study. The heart blood was obtained to measure the serum concentration of ALT, AST, BUN, and creatinine. The kidneys and livers of the animals were harvested and fixed in formalin 10%, paraffinized, sectioned (4 µm), and stained with hematoxylin and eosin for histopathological evaluation by light microscopy [24].

### 2.9. Statistical Analyses

All statistical analyses were performed using GraphPad Prism software version 8.00. The results of size, zeta potential, drug encapsulation efficiency, drug release, cell viability, ROS, mortality rate, weight changes, renal blood factors, and liver blood enzymes were expressed as the mean ± standard deviation (SD, *n* = 3). Statistical differences were analyzed by one-way analysis of variance (ANOVA) test. Statistical analysis was carried out using nonlinear regression analysis, and comparisons were made for IC_50_ values utilizing Tukey’s test.

## 3. Results and Discussion

### 3.1. Nanoparticle Characterization

Both the PEGylated and non-PEGylated nanoparticles were successfully synthesized using the reverse-phase evaporation method. The nanoparticles were characterized in terms of size, PDI, and zeta potential. The results of the Zetasizer instrument demonstrated that both the PEGylated and non-PEGylated nanoparticles were synthesized at the nanoscale size (Figure S1, Table 2). The size of Lip, Lip-DOX, Lip-CB, and Lip-DOX/CB were 195 ± 9, 223 ± 10, 232 ± 11, and 225 ± 11 nm, respectively. These formulations also demonstrated negative zeta potential (−28 ± 1.4, −25 ± 1.3, −21 ± 1, −18 ± 0.8 mV for Lip, Lip-DOX, Lip-CB, and Lip-DOX/CB, respectively), which made them repel each other, preventing their aggregation. In addition, all these formulations demonstrated PDI values in the range of 0.1–0.4 (0.368 ± 0.016, 0.344 ± 0.015, 0.298 ± 0.013, 0.262 ± 0.01 nm for Lip, Lip-DOX, Lip-CB, and Lip-DOX/CB, respectively), confirming that all of them were monodisperse and homogeneous [25,26]. To improve the blood half-life and biocompatibility [20,27], solubility, the efficiency of tumor targeting [11,12,13,19], profile of drug release [28], stability [29,30], and oral absorption [31], the nanoparticles were PEGylated. PEG functions as a lamellarity reducing agent and improves stability, which prevents nanoparticle aggregation [32,33,34].

**Figure 1:**
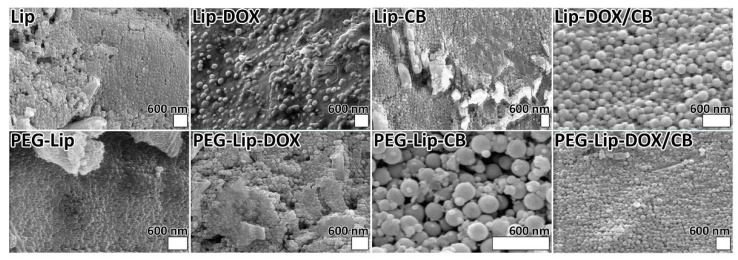
Scanning electron microscopy (SEM) of Lip, Lip-DOX, Lip-CB, Lip-DOX/CB, PEG-Lip, PEG-Lip-DOX, PEG-Lip-CB, and PEG-Lip-DOX/CB.

The PEGylated nanoparticles (PEG-Lip, PEG-Lip-DOX, PEG-Lip-CB, and PEG-Lip-DOX/CB) were smaller than their non-PEGylated counterparts (Table 2). They (PEG-Lip, PEG-Lip-DOX, PEG-Lip-CB, and PEG-Lip-DOX/CB) also demonstrated lower PDI values than the non-PEGylated nanoparticles. The lower size and PDI values of PEGylated liposomes, compared to non-PEGylated liposomes (~80 nm vs. ~100 nm), were also reported previously [33]. Nanoparticles with a size below 300 nm could efficiently internalize into target cells and, as a result, increase the intracellular concentration of their cargo [19,35,36]. This could result from the ability of PEG to reduce lamellarity and increase the stability of conjugates [33,34]. PEG could increase the intensity of lateral repulsion toward the lipid bilayers that causes a curve in the lipid bilayer, leading to a decrease in the size of the vesicle [33]. In addition, PEG as a stabilizing agent [34] causes an inhibition in nanoparticle aggregation [37], resulting in a lower PDI value and a further size decrease in the PEGylated liposomes [38,39]. The zeta potential of the PEGylated nanoparticles, compared to the non-PEGylated nanoparticles, also increased. The zeta potential differences between the PEGylated and non-PEGylated nanoparticles confirmed the successful PEGylation of the nanoparticles. In addition, the morphology of the nanoparticles was evaluated and compared using an SEM instrument. The results of the SEM demonstrated that monodisperse and homogeneous spherical nanoparticles with smooth surfaces were formed (Figure 1). The nanoparticles were formed almost without apparent aggregation, confirming that the particles could redisperse after freeze-drying. In addition, the monodispersibility of the nanoparticles was confirmed by measuring the PDI values of the nanoparticles (Table 3).

The EE% and LE% of the DOX-loaded, CB-loaded, and DOX + CB-loaded nanoparticles were also measured spectrophotometrically at 235 and 480 nm. The EE% and LE% for Lip-DOX, Lip-CB, and Lip-DOX/CB were found to be 84 and 25.2, 87.1 and 26.1, and 81.3 and 12.4%, respectively. These values for the PEGylated nanoparticles increased (88.1 and 22.1, 90.1 and 22.6, and 83.9 and 10.7%, respectively). This might have resulted from the function of PEG, which can change the hydrophobicity, rigidity, chain order and spacing between tails of the lipid membrane, resulting in an improvement in the EE% [40]. These results were in agreement with the results of the Sacchetti et al. study [41], in which PEGylated liposomes, compared to non-PEGylated liposomes, demonstrated a higher EE% (38 vs. 36%).

### 3.2. Release Study

**Figure 2:**
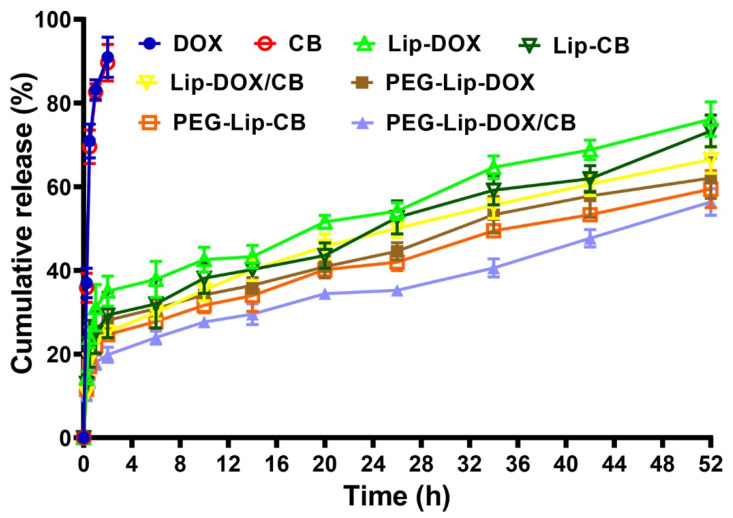
Cumulative drug release from DOX and CB solutions, Lip-DOX, Lip-CB, Lip-DOX/CB, PEG-Lip-DOX, PEG-Lip-CB, and PEG-Lip-DOX/CB. As the figure shows, the amount of drug release from the PEGylated nanoparticles, compared to their non-PEGylated counterparts, was significantly lower (*p* < 0.05). Results are expressed as mean ± SD of three independent experiments.

The efficacy of PEG-Lip-DOX, PEG-Lip-CB, and PEG-Lip-DOX/CB, compared to Lip-DOX, Lip-CB, and Lip-DOX/CB, in controlling the release of the loaded drugs was evaluated at pH 7.4 using a dialysis bag method and standard curves (Figure S2). The results demonstrated that the loaded drug was released from the formulations in a biphasic manner, in which the release was initiated with a burst phase and continued with a slower sustained release. In the burst phase, 24, 22.5, 18.5, 19, 17, and 15% of the loaded drugs were released from Lip-DOX, Lip-CB, Lip-DOX/CB, PEG-Lip-DOX, PEG-Lip-CB, and PEG-Lip-DOX/CB, respectively, in the first 30 min of the study. These burst releases could have resulted from the release of the adsorbed drug [19]. These burst releases continued with a slow and sustained release, in which 76, 73.3, 66.5, 62.1, 59.6, and 56.3% of the loaded drugs were released from Lip-DOX, Lip-CB, Lip-DOX/CB, PEG-Lip-DOX, PEG-Lip-CB, and PEG-Lip-DOX/CB, respectively, after 52 h (Figure 2). According to these results, the PEGylated formulations (PEG-Lip-DOX, PEG-Lip-CB, PEG-Lip-DOX/CB), compared to the non-PEGylated formulations (Lip-DOX, Lip-CB, and Lip-DOX/CB), were more efficient by ~18.3, 18.7, and 15.4%, respectively, in preserving the loaded drugs and releasing them for a longer time. PEG, through coating the surface of nanoparticles, restrains the drug leakage from the particles. Premature drug release is a problem for the application of nanoparticles that could be resolved through PEGylation. In addition, the results demonstrated that 89–93 and 100% of the standard DOX and CB were released from their relevant solutions after 2 and 2.5 h, respectively (Figure 2), indicating the high efficacy of PEG-Lip-DOX, PEG-Lip-CB, PEG-Lip-DOX/CB, Lip-DOX, Lip-CB, and Lip-DOX/CB to control the drug release. Papp et al. [42] also demonstrated that reversed-phase-synthesized PEGylated liposomes, compared to reversed-phase-synthesized non-PEGylated liposomes, caused a lower amount of drug release after 48 h (~20 vs. ~50%). 

The kinetics of drug release from Lip-DOX, Lip-CB, Lip-DOX/CB, PEG-Lip-DOX, PEG-Lip-CB, and PEG-Lip-DOX/CB was also measured using various mathematical models, including zero order, first order, Higuchi, and Korsmeyer–Peppas. In the zero-order kinetic model, the rate of drug release from the polymer matrix is constant throughout an experiment and is not influenced by the drug concentration. In other words, the same amount of drugs per unit of time is released over time. However, in the first-order kinetic model, the rate of drug release is influenced by the drug concentration and, thus, is reduced with time [43]. In the Higuchi kinetic model, the cumulative amount of the released drug is directly proportional to the square root of time [44], and in the Korsmeyer–Peppas model, the drug release rate is regulated by the rate of diffusion and swelling [45,46]. In the present study, the results demonstrated that Lip-DOX, Lip-CB, Lip-DOX/CB, PEG-Lip-DOX, PEG-Lip-CB, and PEG-Lip-DOX/CB were well-fitted to the Higuchi model with correlation coefficient (R^2^) values of 0.7681, 0.792, 0.8431, 0.7654, 0.8062, and 0.7522, respectively (Figure S3, Tables S1–S6).

### 3.3. Cell Viability Study

**Figure 3:**
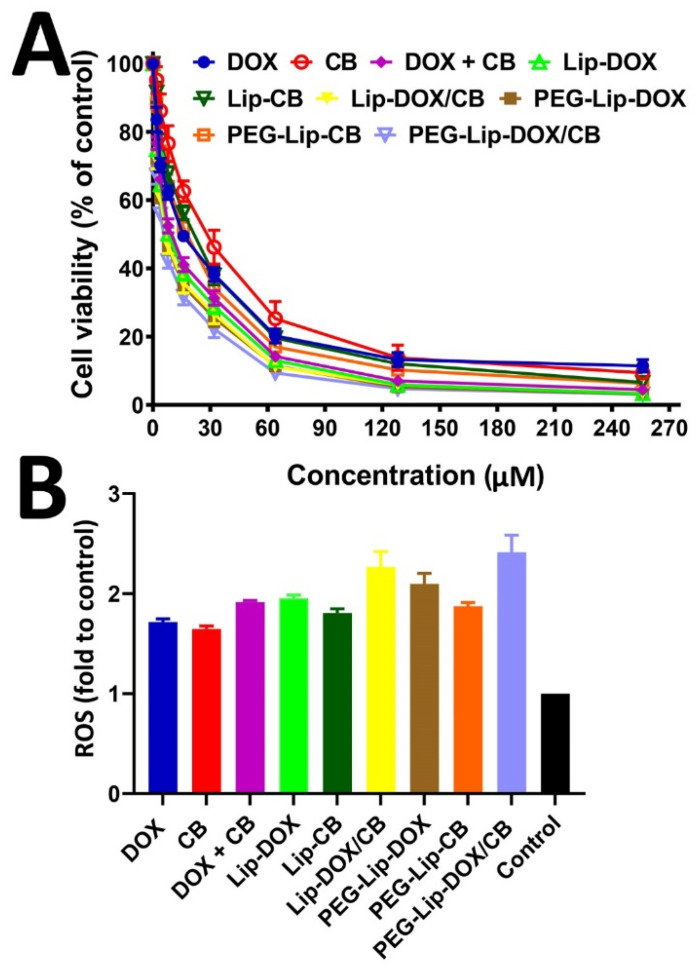
(**A**) Cell viability effects of DOX, CB, Lip-DOX, Lip-CB, Lip-DOX/CB, PEG-Lip-DOX, PEG-Lip-CB, and PEG-Lip-DOX/CB against rat glioma C6 cells, measured by MTT assay. (**B**) Intracellular generation of reactive oxygen species (ROS) in C6 cells after incubation with DOX, CB, DOX + CB, Lip-DOX, Lip-CB, Lip-DOX/CB, PEG-Lip-DOX, PEG-Lip-CB, and PEG-Lip-DOX/CB. Incubating C6 cells with the formations caused cell damage by producing intracellular ROS and oxidative stress. PEG-Lip-DOX, PEG-Lip-CB, and PEG-Lip-DOX/CB, compared to Lip-DOX, Lip-CB, and Lip-DOX/CB, were more potent in producing intracellular ROS. In addition, DOX + CB, compared to DOX and CB, was more potent in producing intracellular ROS, indicating the synergy between the drugs. The data are expressed as mean ± SD (*n* = 3).

Nanoparticles, as drug carriers, are able to improve the therapeutic effects of drugs [19,26,47]. Nanoparticles can increase the drug concentration in tumor cells by increasing the drug circulation time [19]. They can deliver drugs to the target sites in vivo and consequently inhibit the drug side effects on normal cells [19]. In this study, the efficacy of DOX, CB, DOX + CB, Lip-DOX, Lip-CB, Lip-DOX/CB, PEG-Lip-CB, PEG-Lip-DOX, and PEG-Lip-DOX/CB on rat glioma C6 cells were evaluated and compared using an MTT assay. First, it was found that Lip and PEG-Lip were safe and non-toxic at a concentration of 40 mg/mL. The results demonstrated that DOX, CB, DOX + CB, Lip-DOX, Lip-CB, Lip-DOX/CB, PEG-Lip-CB, PEG-Lip-DOX, and PEG-Lip-DOX/CB decreased the viability of the C6 cells in a concentration-dependent manner, in which, by increasing the drug concentration, the cell viability decreased (Figure 3A). In addition, the results demonstrated that the encapsulation of DOX and CB into Lip and PEG-Lip caused an increase in the cytotoxicity effects of the drugs (IC_50_: 16.6, 25.4, 12.9, 12.3, 20.1, 10.1, 10.9, 17.3, and 8.7 µM for DOX, CB, DOX + CB, Lip-DOX, Lip-CB, Lip-DOX/CB, PEG-Lip-DOX, PEG-Lip-CB, and PEG-Lip-DOX/CB, respectively). However, PEG-Lip, compared to Lip, caused a higher drug cytotoxicity. This could be due to the pattern of drug release, smaller size, and higher EE% of PEG-Lip compared to Lip. These results were in agreement with the results of the Najlah et al. study [33], where disulfiram-loaded PEGylated liposomes, compared to disulfiram-loaded non-PEGylated liposomes, caused higher cytotoxicity by 8.5 and 22% against human colorectal cancer H630 WT- and 5FU-resistant human colorectal cancer H630 R10 cells, respectively. In addition, Lip-DOX/CB and PEG-Lip-DOX/CB, compared to Lip-DOX, Lip-CB, PEG-Lip-DOX, and PEG-Lip-CB, demonstrated higher cytotoxicity effects against C6 cells, indicating the synergy between DOX and CB. In addition, the combination of DOX and CB compared to their individual forms caused higher cytotoxicity effects, confirming the synergy between the drugs.

### 3.4. Stability Study

**Figure 4:**
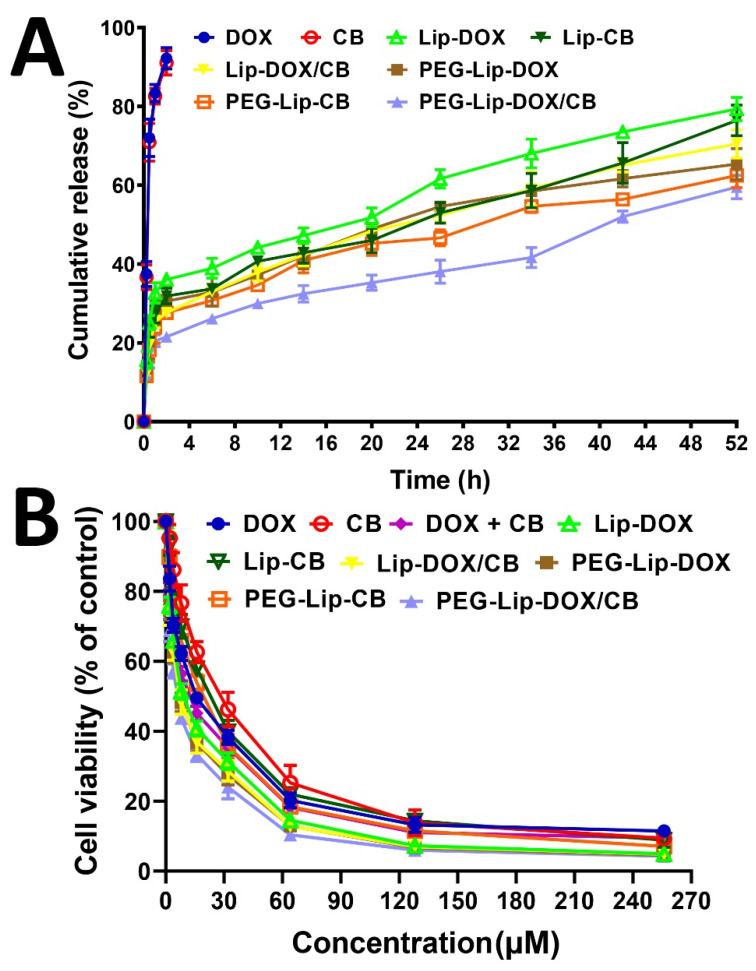
(**A**) Cumulative drug release from Lip-DOX, Lip-CB, Lip-DOX/CB, PEG-Lip-DOX, PEG-Lip-CB, and PEG-Lip-DOX/CB and (**B**) cell viability effects of Lip-DOX, Lip-CB, Lip-DOX/CB, PEG-Lip-DOX, PEG-Lip-CB, and PEG-Lip-DOX/CB three months after preparation. The data are expressed as mean ± SD (*n* = 3).

Nanoparticle stability has a key role in the synthesis and storage of nanoparticles and their efficacy in the delivery of therapeutics in blood circulation [48,49]. Nanoparticles must be highly stable to preserve their cargos under the rough conditions of biological environments, such as low pH, enzymatic decomposition, and vascular endothelial obstacles [50]. Thus, the stability of Lip-DOX, Lip-CB, Lip-DOX/CB, PEG-Lip-DOX, PEG-Lip-CB, and PEG-Lip-DOX/CB was measured and compared three months after their synthesis in terms of drug release, in vitro antitumor effects, and drug loading efficiency, and the results were compared with those obtained at the production time. The results of the drug release study demonstrated that Lip-DOX, Lip-CB, Lip-DOX/CB, PEG-Lip-DOX, PEG-Lip-CB, and PEG-Lip-DOX/CB preserved their efficacy in controlling the release of their cargoes, in which they released 79.3, 76.7, 69.3, 65.3, 62.4, and 59.5% of the loaded drugs, respectively, after 52 h (Table 4, Figure 4A), which was not significantly different from the results obtained at the production time.

In addition, the results of cell viability were not significantly changed compared to the results obtained at the production time, in which the IC_50_ values for Lip-DOX, Lip-CB, Lip-DOX/CB, PEG-Lip-DOX, PEG-Lip-CB, and PEG-Lip-DOX/CB were 13.7, 22.6, 12.4, 12.1, 18.9, and 9.6 µM, respectively, while these values at the production time were 12.3, 20.1, 10.8, 10.9, 17.3, and 8.7 µM, respectively (Figure 4B). In addition, the values of LE% measured three months after the production time were comparable to those obtained at the production time, in which LE% for Lip-DOX, Lip-CB, Lip-DOX/CB, PEG-Lip-DOX, PEG-Lip-CB, and PEG-Lip-DOX/CB was 24.3, 25.4, 11.5, 21.5, 22, and 10.2%, respectively, while these values at the production time were 25.2, 26.1, 11.5, 22.1, 22.6, and 10.7%, respectively. Based on these results, Lip and PEG-Lip were stable carriers for DOX and CB. In addition, the stability of Lip-DOX, Lip-CB, Lip-DOX/CB, PEG-Lip-DOX, PEG-Lip-CB, and PEG-Lip-DOX/CB was evaluated by measuring the size and PDI of the formulations, and the results demonstrated that the size of Lip-DOX, Lip-CB, Lip-DOX/CB, PEG-Lip-DOX, PEG-Lip-CB, and PEG-Lip-DOX/CB did not significantly increase after three months (Table 5). In addition, the results showed that the PEGylated nanoparticles (i.e., PEG-Lip-DOX, PEG-Lip-CB, and PEG-Lip-DOX/CB), compared to their non-PEGylated counterparts (Lip-DOX, Lip-CB, and Lip-DOX/CB), were more potent in maintaining their size. In addition, the results of serum stability were in agreement with the results of the size and PDI measurements, in which the PEGylated nanoparticles, compared to their non-PEGylated counterparts, had a higher serum stability (11.9% size increment for the PEGylated nanoparticles, compared to 19.2% size increment for the non-PEGylated nanoparticles, Figure S4). Ramana et al. [51] also demonstrated that drug-loaded PEGylated liposomes, compared to drug-loaded non-PEGylated liposomes, were more stable, in which a lower reduction (25 vs. 79%) in the zeta potential values was observed for the drug-loaded PEGylated liposomes, compared to that of drug-loaded non-PEGylated liposomes, 6 h after incubation in the protein medium. 

### 3.5. Reactive Oxygen Species Assay

ROS (e.g., superoxide radicals (O_2_•^−^) and peroxides (O_2_^2−^)) are highly reactive, and their excessive amounts can induce oxidative damage to cellular components such as lipids, enzymes, and DNA [52]. Normal cells, through a series of antioxidant systems, equilibrate ROS levels and usually maintain redox homeostasis [52], while cancer cells, owing to the disruption of redox homeostasis and stress adaptation, are more susceptible to oxidative stress damage [52]. Most chemotherapeutics can cause an increase in the intracellular concentrations of ROS [53] and, as a result, cancer cell death. In this study, the efficacy of Lip-DOX, Lip-CB, Lip-DOX/CB, PEG-Lip-DOX, PEG-Lip-CB, and PEG-Lip-DOX/CB in producing intracellular ROS in rat glioma C6 cells was measured and compared using a DCFH-DA probe. The results demonstrated that DOX, CB, DOX + CB, Lip-DOX, Lip-CB, Lip-DOX/CB, PEG-Lip-DOX, PEG-Lip-CB, and PEG-Lip-DOX/CB, compared to the control group, were more efficient in producing intracellular ROS by 1.7-, 1.6-, 1.9-, 1.9-, 1.8-, 2.3-, 2.1-, 1.9-, and 2.4-fold, respectively (Figure 3B). The higher efficacy of the PEGylated nanoparticles, compared to the non-PEGylated nanoparticles, in producing intracellular ROS could be due to the higher EE%, smaller size, and the pattern of drug release from the PEGylated nanoparticles. In addition, DOX, in combination with CB, compared to their individual forms, caused a higher potency in producing ROS, indicating the synergy between the drugs. These results were in agreement with the results of cell viability, in which the PEGylated nanoparticles compared to the non-PEGylated nanoparticles and the combination of DOX + CB compared to their individual forms, caused a higher efficacy in producing intracellular ROS (Figure 3B).

### 3.6. Animal Study

**Figure 5:**
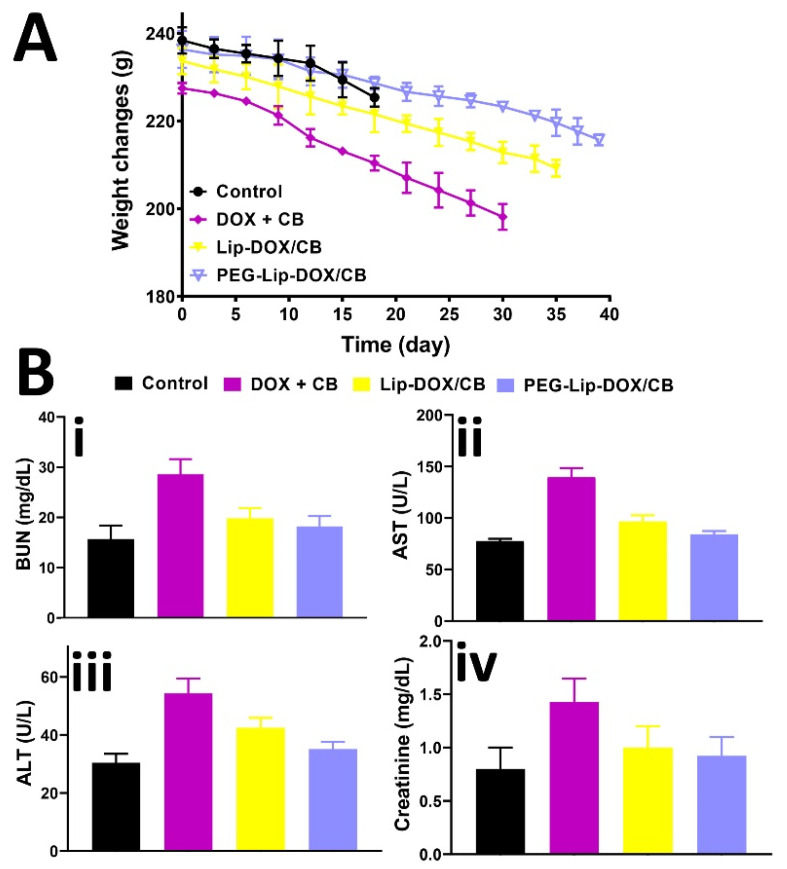
(**A**) Weight changes in brain-tumor-bearing rats in DOX + CB, Lip-DOX/CB, and PEG-Lip-DOX/CB receiver groups at three-day intervals and for 39 days after tumor cell inoculation and (**B**) serum concentrations of (**i**) alanine aminotransferase (ALT), (**ii**) aspartate aminotransferase (AST), (**iii**) BUN, and (**iv**) creatinine in DOX + CB, Lip-DOX/CB, and PEG-Lip-DOX/CB receiver glioblastoma-bearing rats compared to the control group.

**Figure 6:**
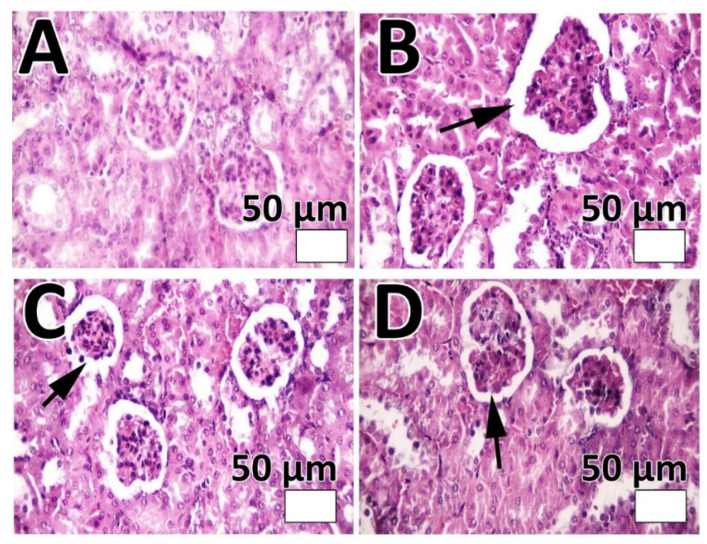
Results of renal histopathological studies in (**A**) control; (**B**) DOX + CB; (**C**) Lip-DOX/CB; and (**D**) PEG-Lip-DOX/CB receiver groups of glioblastoma-bearing rats. As the results show, acute tubular necrosis was more evident in DOX + CB group, compared to Lip-DOX/CB and PEG-Lip-DOX/CB groups. Arrows show acute tubular necrosis (Magnification ×40).

**Figure 7:**
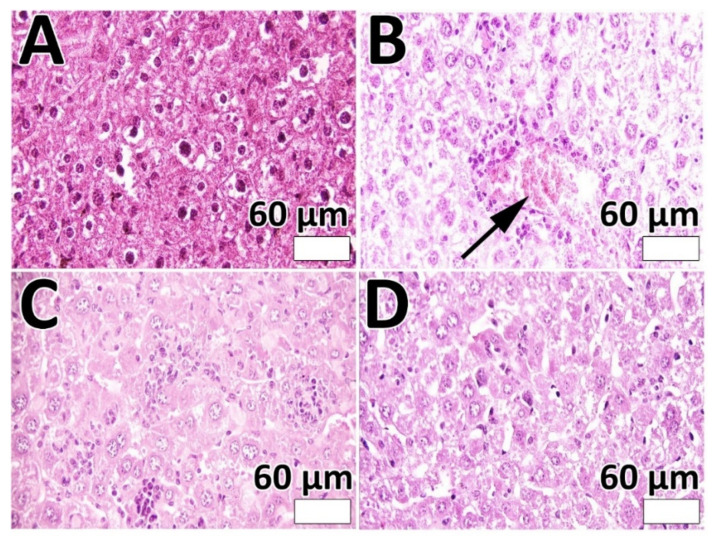
Results of liver histopathological studies in (**A**) control; (**B**) DOX + CB; (**C**) Lip-DOX/CB; and (**D**) PEG-Lip-DOX/CB receiver groups. As the results show, no cellular damage was observed in control, Lip-DOX/CB, and PEG-Lip-DOX/CB receiver groups; however, liver cell necrosis (arrow) was observed in DOX + CB group. (Magnification ×40).

The efficacy of PEG-Lip-DOX/CB and Lip-DOX/CB, compared to DOX + CB, in the treatment of glioblastoma-bearing rats was measured using rat glioma C6 cells. These cells are extensively used as a model of glioblastoma. The profiles of gene expression and histopathological features of these cells are almost the same as human glioblastoma [54]. The cells are highly invasive with rapid division and demonstrate features of metastasized brain tumor cells [55]. The features of tumor development were demonstrated only 8 days after tumor cells inoculation, including decreasing the physical activity, alertness, and responses to contact. In addition, the ability of the animals to clean themselves was decreased. The systemic toxicity of the formulations was evaluated by measuring the weight loss and serum level of ALT, AST, BUN, and creatinine. The results demonstrated that DOX + CB, Lip-DOX/CB, and PEG-Lip-DOX/CB caused a weight loss of 13, 10.5, and 8.7%, respectively (Figure 5A). In addition, the results demonstrated that PEG-Lip-DOX/CB compared to Lip-DOX/CB and DOX + CB caused a lesser increase in the serum level of ALT, AST, BUN, and creatinine (Figure 5B, ALT: 30.5 ± 1.6, 54 ± 2.6, 42.5 ± 2.1, and 35.2 ± 1.8 U/L; AST: 77.4 ± 3.6, 139.6 ± 6.3, 96.7 ± 4.7, and 87.2 ± 4.2 U/L; BUN: 15.7 ± 0.8, 28.6 ± 1.4, 19.9 ± 0.9, and 18.2 ± 0.9 mg/dL; creatinine: 0.8 ± 0.04, 1.44 ± 0.7, 1 ± 0.4, 0.92 ± 0.05 mg/dL in the control, DOX + CB, Lip-DOX/CB, and PEG-Lip-DOX/CB receiver groups, respectively), indicating the efficacy of PEG-Lip-DOX/CB compared to the standard drugs and Lip-DOX/CB in decreasing the side effects of DOX and CB. The toxicity was also evaluated by histopathological studies. The results demonstrated that PEG-Lip-DOX/CB, compared to Lip-DOX/CB and DOX + CB, was more efficient in decreasing the severity of acute tubular necrosis (Table 5, Figure 6) and liver cell necrosis (Table 6, Figure 7). This could have resulted from the higher stability of PEG-Lip-DOX/CB in blood circulation and the pattern of drug release from these nanoparticles, which release the drugs for a longer time [56]. Overall, the surface modification of nanoparticles with a neutral polymer, such as PEG, can cause (i) a reduction in immunogenicity and toxicity, (ii) an alteration in the biodistribution, (iii) an increase in the blood circulation time [57] and the tumor accumulation of nanoparticles [58], leading to an increment in the therapeutic effects of the conjugate [59]. In addition, PEGylation enhances the aqueous solubility of sparingly soluble drugs, increases the drug stability, and causes an increase in the time of drug release and retention time of drug conjugates in vivo. Thus, PEGylation can improve the drug efficacy and stability and decrease the dosing frequency of drug products [60].

In addition, the efficacy of the formulations in increasing the survival of brain-tumor-bearing rats was evaluated. The results demonstrated that all of the brain-tumor-bearing rats in the control group were dead by day 18, while the animals in the DOX + CB, Lip-DOX/CB, and PEG-Lip-DOX/CB groups were dead by days 30, 35, and 39, respectively. These results indicated the efficacy of PEG-Lip-DOX/CB, compared to DOX + CB and Lip-DOX/CB, in improving the effectiveness of the drugs in treating a brain tumor. These results were in agreement with the results of the study by Shamshiri et al. [61], where interferon-gamma (IFN-γ)-loaded PEGylated liposomes, compared to IFN-γ-loaded non-PEGylated liposomes, caused a significant decrease in the tumor volume in a mouse model of C26 colon cancer by ~1.4-fold (~530 vs. ~770 mm^3^) on day 30 after tumor cell inoculation.

## 4. Conclusions

This study aimed to improve the efficacy of DOX + CB for the treatment of brain tumors using a PEGylated nanoformulation of the drugs. The nanoformulation was successfully synthesized with a size of 212 ± 10 nm and LE% of 10.65%, which could release the drugs in a controlled manner (56.25% of the loaded drugs after 52 h). In addition, PEG-Lip-DOX/CB could significantly increase the efficacy of the loaded drugs in terms of cytotoxicity (by 1.5-fold) and ROS production (by 1.3-fold). PEG-Lip-DOX/CB, compared to DOX + CB and Lip-DOX/CB, could cause a significant increase in the efficacy of the loaded drug in the treatment of glioblastoma-bearing rats, in which PEG-Lip-DOX/CB could increase the survival time of the animals by 23.1% and 10.2%, compared to DOX + CB and Lip-DOX/CB, respectively. The efficacy of PEG-Lip-DOX/CB in decreasing the side effects of the drugs was also evaluated, and the results demonstrated that PEG-Lip-DOX/CB, compared to DOX + CB and Lip-DOX/CB, was 1.5- and 1.2-fold more potent, respectively, in inhibiting the weight loss of the animals. These results were confirmed by the results of histopathological studies, in which PEG-Lip-DOX/CB, compared to DOX + CB and Lip-DOX/CB, caused less severe acute tubular necrosis and liver cell necrosis. It should be noted that the co-delivery of DOX and CB for cancer treatment has not been reported until now, and this seems to be the first study that has evaluated the efficacy of DOX and CB as a nanoplatform for the treatment of brain tumor. Overall, the results of this study demonstrated that PEG-Lip could be considered a promising carrier for the co-delivery of DOX and CB for the treatment of glioblastoma. 

## Figures and Tables

**Table 1:** Amount of source ingredients used to synthesize the formulations.

	Lecithin		Cholesterol		DSPE-PEG2000		CB		DOX	
	mg	mM	mg	mM	mg	mM	mg	mM	mg	mM
Lip	21.6	4.0	8	2.9	N/A	N/A	N/A	N/A	N/A	N/A
PEG-Lip	21.6	4.0	8	2.9	7.3	361.4	N/A	N/A	N/A	N/A
Lip-CB	21.6	4.0	8	2.9	N/A	N/A	12	4.5	N/A	N/A
PEG-Lip-CB	21.6	4.0	8	2.9	7.3	361.4	12	4.5	N/A	N/A
Lip-DOX	21.6	4.0	8	2.9	N/A	N/A	N/A	N/A	12	2.9
PEG-Lip-DOX	21.6	4.0	8	2.9	7.3	361.4	N/A	N/A	12	2.9
Lip-DOX/CB	21.6	4.0	8	2.9	N/A	N/A	6.0	2.2	6	1.4
PEG-Lip-DOX/CB	21.6	4.0	8	2.9	7.3	361.4	6.0	2.2	6	1.4

N/A: Not applicable.

**Table 2:** Size, polydispersity index (PDI), zeta potential, EE%, and LE% of various nanoparticles obtained by Zetasizer and spectrophotometer instruments.

	Size (nm)	PDI	Zeta Potential (mV)	EE%	LE%
Lip	195 ± 9	0.368 ± 0.016	−28 ± 1.4	N/A	N/A
PEG-Lip	180 ± 8	0.323 ± 0.016	−23 ± 1.1	N/A	N/A
Lip-CB	232 ± 11	0.298 ± 0.013	−21 ± 1	87.09	26.1
PEG-Lip-CB	218 ± 11,	0.286 ± 0.013	−17 ± 0.8	90.1	22.6
Lip-DOX	223 ± 10	0.344 ± 0.015	−25 ± 1.3	84.0	25.2
PEG-Lip-DOX	208 ± 11	0.302 ± 0.015	−20 ± 1.0	88.1	22.1
Lip-DOX/CB	225 ± 11	0.262 ± 0.01	−18 ± 0.8	78.3/84.3	11.9/12.8
PEG-Lip-DOX/CB	212 ± 10	0.211 ± 0.01	−13 ± 0.6	83.9	10.7

N/A: Not applicable.

**Table 3:** PDI values of the nanoparticles after freeze-drying obtained by the Zetasizer instrument.

	PDI
Lip	0.492 ± 0.019
PEG-Lip	0.418 ± 0.018
Lip-CB	0.431 ± 0.020
PEG-Lip-CB	0.375 ± 0.018
Lip-DOX	0.496 ± 0.021
PEG-Lip-DOX	0.413 ± 0.019
Lip-DOX/CB	0.408 ± 0.018
PEG-Lip-DOX/CB	0.316 ± 0.019

**Table 4:** The values of drug release, cell viability, and LE for Lip-DOX, Lip-CB, Lip-DOX/CB, PEG-Lip-DOX, PEG-Lip-CB, and PEG-Lip-DOX/CB at the preparation time and three months after preparation.

	Drug Release (%)		Cell Viability (IC_50_)		LE%	
	P.T	T.M.P.T	P.T	T.M.P.T	P.T	T.M.P.T
Lip-CB	73.3	76.7	20.1	22.6	26.1	25.4
PEG-Lip-CB	59.6	62.4	17.3	18.9	22.6	22
Lip-DOX	76	79.3	12.3	13.7	25.2	24.3
PEG-Lip-DOX	62.1	65.3	10.9	12.1	22.1	21.5
Lip-DOX/CB	66.5	69.6	10.8	12.4	11.5	11.5
PEG-Lip-DOX/CB	56.25	59.5	8.7	9.6	10.65	10.2

P.T: Preparation time; T.M.P.T: three months after the preparation time.

**Table 5:** Size and polydispersity index (PDI) for Lip-DOX, Lip-CB, Lip-DOX/CB, PEG-Lip-DOX, PEG-Lip-CB, and PEG-Lip-DOX/CB on the production time (P.T) and three months after preparation (T.M.P.T).

	Size (nm)		Polydispersity Index (PDI)	
	P.T	T.M.P.T	P.T	T.M.P.T
Lip-CB	232 ± 11	256 ± 12	0.298 ± 0.013	0.326 ± 0.014
PEG-Lip-CB	218 ± 11	232 ± 12	0.286 ± 0.013	0.280 ± 0.011
Lip-DOX	223 ± 10	249 ± 11	0.344 ± 0.015	0.373 ± 0.017
PEG-Lip-DOX	208 ± 11	223 ± 11	0.302 ± 0.015	0.331 ± 0.017
Lip-DOX/CB	225 ± 11	249 ± 12	0.262 ± 0.01	0.312 ± 0.03
PEG-Lip-DOX/CB	212 ± 10	227 ± 11	0.211 ± 0.01	0.232 ± 0.03

P.T: Preparation time; T.M.P.T: Three months after the preparation time.

**Table 6:** Histological evaluation of organ toxicity in brain-tumor-bearing rats after treatment with DOX + CB, Lip-DOX/CB, and PEG-Lip-DOX/CB, compared to the control group.

	Number of Animals	Organ	Score
Control	8	Liver	0–1
		Kidney	0–1
DOX + CB	8	Liver	2
		Kidney	1–2
Lip-DOX/CB	8	Liver	0–1
		Kidney	0–1
PEG-Lip-DOX/CB	8	Liver	0–1
		Kidney	0–1

## Data Availability

The data presented in this study are available on request from the corresponding author (H.E.S).

## Supplementary Materials

The following supporting information can be downloaded at: https://www.mdpi.com/article/10.3390/pharmaceutics14102183/s1, Figure S1: Size diagram, polydispersity index (PDI), and zeta potential values of Lip, Lip-DOX, Lip-CB, Lip-DOX/CB, PEG-Lip, PEG-Lip-DOX, PEG-Lip-CB, and PEG-Lip-DOX/CB; Figure S2: The standard curves (drug concentration vs. optical density (OD)) of DOX and CB, respectively; Figure S3: The Higuchi model of DOX and CB release from (A) Lip-DOX, (B) Lip-CB, (C) Lip-DOX/CB, (D) PEG-Lip-DOX, (E) PEG-Lip-CB, and (F) PEG-Lip-DOX/CB. Results are expressed as mean ± SD of three independent experiments; Figure S4: The stability of Lip-DOX, Lip-CB, Lip-DOX/CB, PEG-Lip-DOX, PEG-Lip-CB, and PEG-Lip-DOX/CB in fetal bovine serum (FBS) over 5 h at 37 °C. Data are expressed as mean ± SD (*n* = 3); Table S1: Formulation and optimization of non-PEGylated liposome nanoparticles (Lip) using various lecithin/cholesterol ratios; Table S2: Formulation and optimization of PEGylated liposome nanoparticles using various PEG/(lecithin + cholesterol) lipid ratios; Table S3: Formulation and optimization of drug-loaded PEGylated and non-PEGylated liposome nanoparticles using various DOX/CB concentrations; Table S4: Measured values of cumulative % drug released, % drug remaining, square root time, log cumulative % drug remaining, log time, log cumulative % drug released, % drug released, cube root of % drug remaining (W_t_) and W_0_-W_t_ parameters to determine the drug release kinetics for Lip-DOX. W_0_ and W_t_ are the initial and remaining amount of drug in the pharmaceutical dosage form at times 0 and t, respectively; Table S5: Measured values of cumulative % drug released, % drug remaining, square root time, log cumulative % drug remaining, log time, log cumulative % drug released, % drug released, cube root of % drug remaining (W_t_) and W_0_–W_t_ parameters to determine the drug release kinetics for Lip-DOX. W_0_ and W_t_ are the initial and remaining amount of drug in the pharmaceutical dosage form at times 0 and t, respectively; Table S6: Measured values of cumulative % drug released, % drug remaining, square root time, log cumulative % drug remaining, log time, log cumulative % drug released, % drug released, cube root of % drug remaining (W_t_) and W_0_–W_t_ parameters to determine the drug release kinetics for Lip-DOX. W_0_ and W_t_ are the initial and remaining amount of drug in the pharmaceutical dosage form at times 0 and t, respectively; Table S7: Measured values of cumulative % drug released, % drug remaining, square root time, log cumulative % drug remaining, log time, log cumulative % drug released, % drug released, cube root of % drug remaining (W_t_) and W_0_-W_t_ parameters to determine the drug release kinetics for Lip-DOX. W_0_ and W_t_ are the initial and remaining amount of drug in the pharmaceutical dosage form at times 0 and t, respectively; Table S8: Measured values of cumulative % drug released, % drug remaining, square root time, log cumulative % drug remaining, log time, log cumulative % drug released, % drug released, cube root of % drug remaining (W_t_) and W_0_–W_t_ parameters to determine the drug release kinetics for Lip-DOX. W_0_ and W_t_ are the initial and remaining amount of drug in the pharmaceutical dosage form at times 0 and t, respectively; Table S9: Measured values of cumulative % drug released, % drug remaining, square root time, log cumulative % drug remaining, log time, log cumulative % drug released, % drug released, cube root of % drug remaining (W_t_) and W_0_–W_t_ parameters to determine the drug release kinetics for Lip-DOX. W_0_ and W_t_ are the initial and remaining amount of drug in the pharmaceutical dosage form at times 0 and t, respectively.
