# Supplementary material for: Impact of PEGylated Liposomal Doxorubicin and Carboplatin Combination on Glioblastoma
Source: Pharmaceutics. 2022 Oct 13;14(10):2183. doi: 10.3390/pharmaceutics14102183 (PMC9609487; doi:10.3390/pharmaceutics14102183)
Supplement: Supplementary file 1 [file pharmaceutics-14-02183-s001.zip › pharmaceutics-1936572-supplementary.pdf]

pharmaceutics

MDPI

Supplementary Materials: Impact of PEGylated Liposomal Doxorubicin and Carboplatin Combination on Glioblastoma

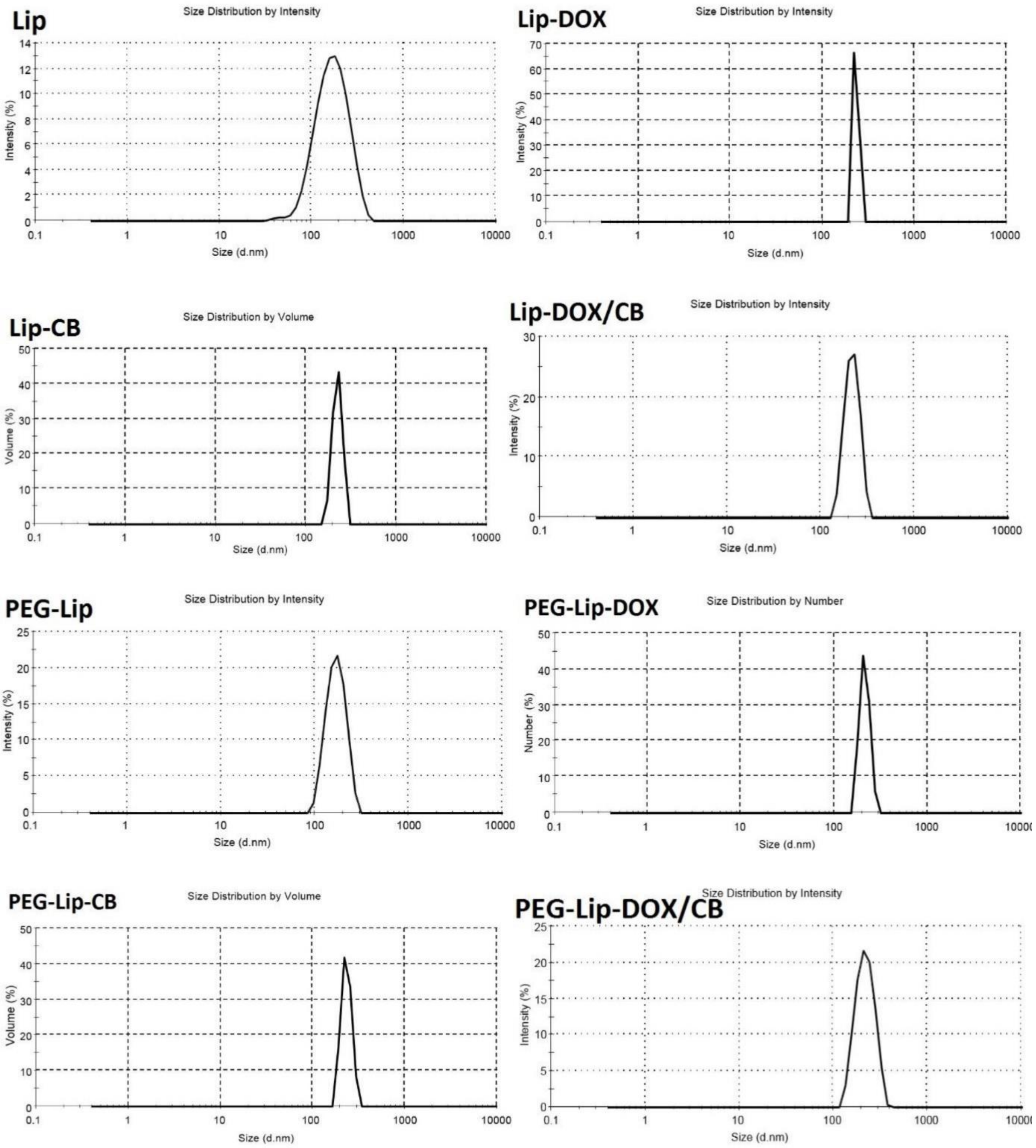

Figure S1. Size diagram, polydispersity index (PDI), and zeta potential values of Lip, Lip-DOX, Lip-CB, Lip-DOX/CB, PEG-Lip, PEG-Lip-DOX, PEG-Lip-CB, and PEG-Lip-DOX/CB.

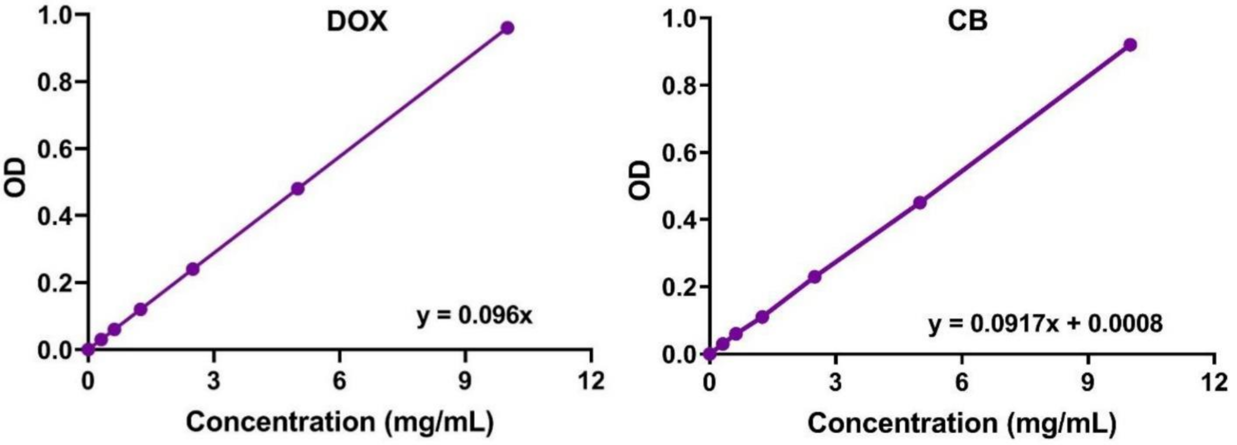

Figure S2. The standard curves (drug concentration vs. optical density (OD)) of DOX and CB, respectively.

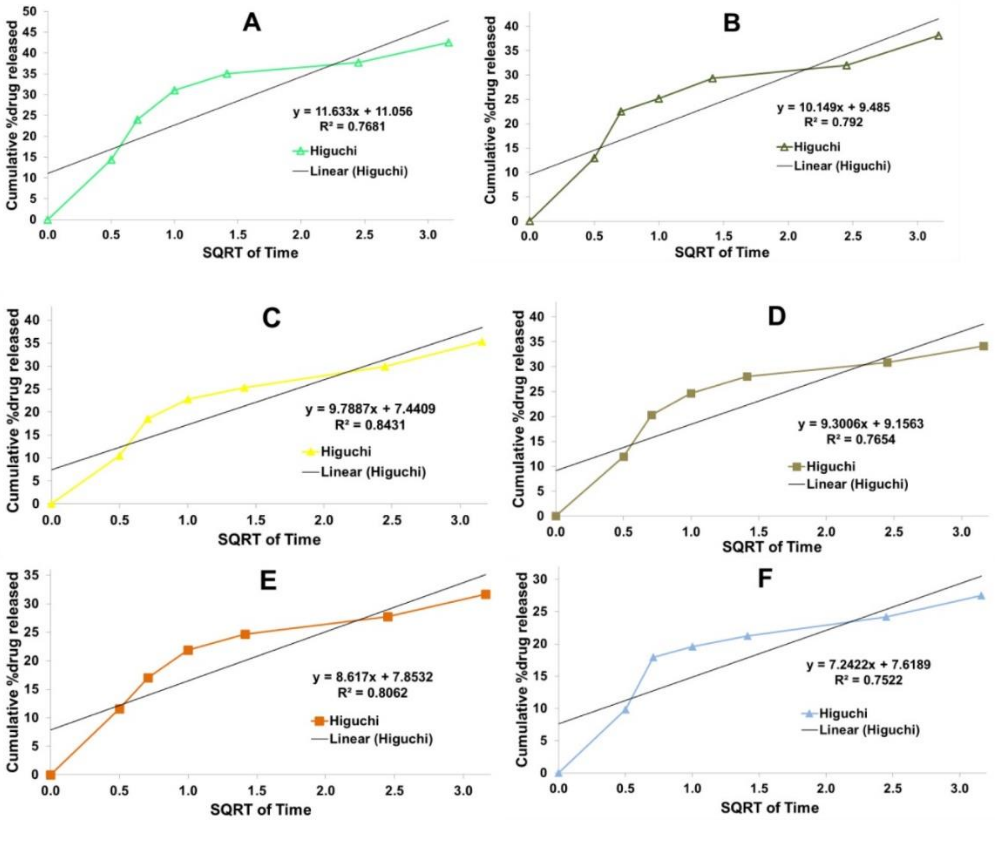

Figure S3. The Higuchi model of DOX and CB release from (A) Lip-DOX, (B) Lip-CB, (C) Lip-DOX/CB, (D) PEG-Lip-DOX, (E) PEG-Lip-CB, and (F) PEG-Lip-DOX/CB. Results are expressed as mean ± SD of three independent experiments.

Figure S4. The stability of Lip-DOX, Lip-CB, Lip-DOX/CB, PEG-Lip-DOX, PEG-Lip-CB, and PEG-Lip-DOX/CB in fetal bovine serum (FBS) over 5 h at 37 °C. Data is expressed as mean ± SD (n = 3).

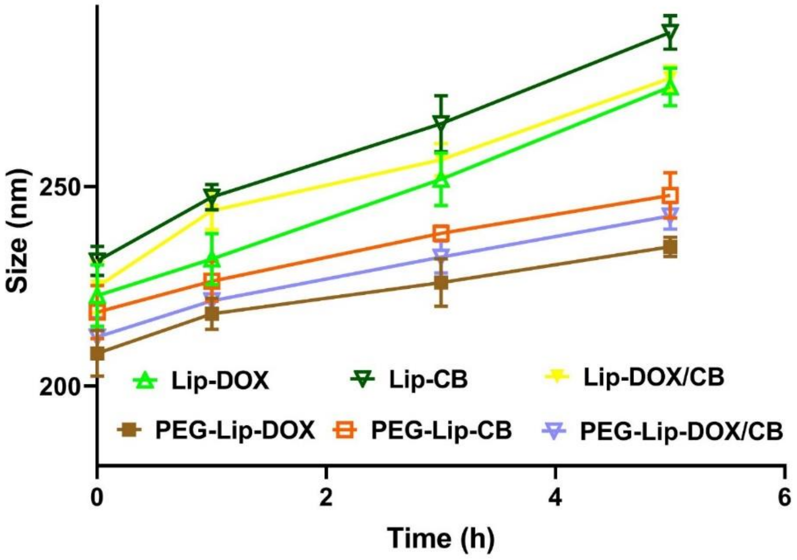

Figure S4. The stability of Lip-DOX, Lip-CB, Lip-DOX/CB, PEG-Lip-DOX, PEG-Lip-CB, and PEG-Lip-DOX/CB in fetal bovine serum (FBS) over 5 h at 37 °C. Data is expressed as mean ± SD (n = 3).

Optimization of the doxorubicin, carboplatin-loaded polyethylene glycol (PEG)ylated liposome (PEG-Lip-DOX/CB) formulation

To optimize the preparation of PEG-Lip-DOX/CB, the formulation composition of liposome nanoparticles (Lip) was optimized in terms of size and polydispersity index (PDI) according to the different weight ratios of lecithin and cholesterol (Table S1). The optimized formulation was selected based on the minimum size and PDI [1, 2]. The optimized formulation with the minimum size and PDI values (Table S1, L3) was then optimized against three different concentrations of DSPE-PEG2000 (Table S2). For this purpose, the three concentrations of DSPE-PEG2000 were mixed with L3 separately, and the optimized concentration with the minimum size and PDI was selected to mix with the three different concentrations of the drugs to find the optimized formulation according to their encapsulation efficiency (EE%, Table S3).

Table S1. Formulation and optimization of non-PEGylated liposome nanoparticles (Lip) using various lecithin/cholesterol ratios.

Code	Lecithin/cholesterol ratio (w/w%)	Size (nm)	PDI

Copyright: © 2022 by the authors. Licensee MDPI, Basel, Switzerland. This article is an open access article distributed under the terms and conditions of the Creative

Commons Attribution (CC BY) license (https://creativecommons.org/licenses/by/4.0/).

**L1**	2.7:2	259.3 ± 12.8	0.337 ± 0.013
**L2**	2.7:1.5	231.1 ± 10.4	0.384 ± 0.018
**L3**	2.7:1	195 ± 9	0.368 ± 0.016
**L4**	5.4:2	289.4 ± 13.7	0.398 ± 0.016
**L5**	5.4:1.5	268.3 ± 11.4	0.378 ± 0.018
**L6**	5.4:1	232.3 ± 11.2	0.377 ± 0.019
**L7**	8.1:2	346.2 ± 15.9	0.463 ± 0.029
**L8**	8.1:1.5	311.8 ± 13.1	0.358 ± 0.026
**L9**	8.1:1	282.1 ± 12.2	0.351 ± 0.022

* PDI: Polydispersity index.

Table S2. Formulation and optimization of PEGylated liposome nanoparticles using various PEG/(lecithin + cholesterol) lipid ratios.

Code	DSPE-PEG2000/lipid ratios (w/w%)	Size (nm)	PDI
**L3P1**	0.25/4	219.2 ± 8.7	0.382 ± 0.018
**L3P2**	0.5/4	204.3 ± 7.7	0.361 ± 0.015
**L3P3**	1/4	180 ± 8	0.323 ± 0.016

Table S3. Formulation and optimization of drug-loaded PEGylated and non-PEGylated liposome nanoparticles using various DOX/CB concentrations.

Code	Drug (w/w%)	Size (nm)	PDI	EE%
**L3C1**	16.9	216.2 ± 9.4	0.333 ± 0.018	72
**L3C2**	23.3	231 ± 11.8	0.339 ± 0.017	76.1
**L3C3**	28.8	225 ± 11	0.262 ± 0.01	81.3
**L3P3C1**	16.9	198.2 ± 10.1	0.369 ± 0.017	73.4
**L3P3C2**	23.3	215.3 ± 11.4	0.249 ± 0.04	77.8
**L3P3C3**	28.8	212 ± 10	0.211 ± 0.01	83.9

*** EE%:** Encapsulation efficiency (%).

1. Gkionis, L., Aojula, H., Harris, L. K., & Tirella, A. (2021). Microfluidic-assisted fabrication of phosphatidylcholine-based liposomes for controlled drug delivery of chemotherapeutics. International journal of pharmaceutics, 604, 120711.

2. Karthika, C., Sureshkumar, R., Upadhyay, D., Janani, S. K., Vasanthi, C., Raja, M., & Upathyayula, S. S. N. (2019). Formulation Development and In vitro Characterization of Solid Self Nano Emulsifying Drug Delivery System for Curcumin to Target Colon Adenocarcinoma. Research Journal of Pharmacy and Technology, 12(7), 3338-3346.

Table S4. Measured values of cumulative % drug released, % drug remaining, square root time, log cumulative % drug remaining, log time, log cumulative % drug released, % drug released, cube root of % drug remaining (W_t) and W_0-W_t parameters to determine the drug release kinetics for Lip-DOX. W_0 and W_t are the initial and remaining amount of drug in the pharmaceutical dosage form at times 0 and t, respectively.

Time (hr)	cumulative % drug released	% drug remaining	Square root time	log Cumu % drug remaining	log time	log Cumu % drug released	% Drug released	Cube Root of % drug Remaining (W_t)	W_0-W_t
0	0	100	0.0	2.0	0.0	0.0	100	4.6	0.0
0.25	14.4	85.6	0.5	1.9	-0.6	1.2	14.4	4.4	0.2
0.5	24	76	0.7	1.9	-0.3	1.4	9.6	4.2	0.4
1.0	31.1	68.9	1.0	1.8	0.0	1.5	7.1	4.1	0.5
2	35.	65.0	1.4	1.8	0.3	1.5	3.9	4.0	0.6
6	37.7	62.3	2.5	1.8	0.8	1.6	2.7	4.0	0.7
10	42.6	57.4	3.2	1.8	1.0	1.6	4.8	3.9	0.8

Table S5. Measured values of cumulative % drug released, % drug remaining, square root time, log cumulative % drug remaining, log time, log cumulative % drug released, % drug released, cube root of % drug remaining (W_t) and W_0-W_t parameters to determine the drug release kinetics for Lip-CB. W_0 and W_t are the initial and remaining amount of drug in the pharmaceutical dosage form at times 0 and t, respectively.

Time (hr)	cumulative % drug released	% drug remaining	Square root time	log Cumu % drug remaining	log time	log Cumu % drug released	% Drug released	Cube Root of % drug Remaining (W_t)	W_0-W_t
0	0	100	0.0	2.0	0.0	0.0	100	4.6	0.0
0.25	13.0	87.0	0.5	1.9	-0.6	1.1	13.0	4.4	0.2
0.5	22.5	77.5	0.7	1.9	-0.3	1.4	9.5	4.3	0.4
1	25.2	74.8	1.0	1.9	0.0	1.4	2.7	4.2	0.4
2	29.3	70. 7	1.4	1.8	0.3	1.5	4.2	4.1	0.5
6	32	68	2.5	1.8	0.8	1.5	2.7	4.1	0.6
10	38.1	61.9	3.2	1.8	1.0	1.6	6.1	4.0	0.7

Table S6. Measured values of cumulative % drug released, % drug remaining, square root time, log cumulative % drug remaining, log time, log cumulative % drug released, % drug released, cube root of % drug remaining (W_t) and W_0-W_t parameters to determine the drug release kinetics for Lip-DOX/CB. W_0 and W_t are the initial and remaining amount of drug in the pharmaceutical dosage form at times 0 and t, respectively.

Time (hr)	cumulative % drug released	% drug remaining	Square root time	log Cumu % drug remaining	log time	log Cumu % drug released	% Drug released	Cube Root of % drug Remaining (W_t)	W_0-W_t
0	0	100	0.0	2.0	0.0	0.0	100	4.6	0.0
0.25	10.5	89.5	0.5	1.9	-0.6	1.0	10.5	4.5	0.2
0.5	18.6	81.4	0.7	1.9	-0.3	1.3	8.0	4.3	0.3
1	22.8	77.2	1.0	1.9	0.0	1.4	4.2	4.3	0.4
2	25.3	74.7	1.4	1.9	0.3	1.4	2.5	4.2	0.4
6	29.9	70.1	2.4	1.8	0.8	1.5	4.6	4.1	0.5
10	35.3	64.7	3.2	1.8	1.0	1.5	5.4	4.0	0.6

Table S7. Measured values of cumulative % drug released, % drug remaining, square root time, log cumulative % drug remaining, log time, log cumulative % drug released, % drug released, cube root of % drug remaining (W_t) and W_0-W_t parameters to determine the drug release kinetics for PEG-Lip-DOX. W_0 and W_t are the initial and remaining amount of drug in the pharmaceutical dosage form at times 0 and t, respectively.

Time (hr)	cumulative % drug released	% drug remaining	Square root time	log Cumu % drug remaining	log time	log Cumu % drug released	% Drug released	Cube Root of % drug Remaining (W_t)	W_0-W_t
0	0	100	0.0	2.0	0.0	0.0	100	4.6	0.0
0.25	11.9	88.1	0.5	1.9	-0.6	1.1	11.9	4.4	0.2
0.5	20.3	79.7	0.7	1.9	-0.3	1.3	8.4	4.3	0.3
1	24.7	75.3	1.0	1.9	0.0	1.4	4.4	4.2	0.4
2	28.1	71.9	1.4	1.9	0.3	1.4	3.4	4.2	0.5
6	30.9	69.1	2.4	1.8	0.8	1.5	2.8	4.1	0.5
10	34.1	65.9	3.2	1.8	1.0	1.5	3.3	4.0	0.6

Table S8. Measured values of cumulative % drug released, % drug remaining, square root time, log cumulative % drug remaining, log time, log cumulative % drug released, % drug released, cube root of % drug remaining (W_t) and W_0-W_t parameters to determine the drug release kinetics for PEG-Lip-CB. W_0 and W_t are the initial and remaining amount of drug in the pharmaceutical dosage form at times 0 and t, respectively.

Time (hr)	cumulative % drug released	% drug remaining	Square root time	log Cumu % drug remaining	log time	log Cumu % drug released	% Drug released	Cube Root of % drug Remaining (W_t)	W_0-W_t
0	0	100	0.0	2.0	0.0	0.0	100	4.6	0.0
0.25	11.6	88.4	0.5	1.9	-0.6	1.1	11.6	4.5	0.2
0.5	17	83	0.7	1.9	-0.3	1.2	5.4	4.4	0.3
1	21.9	78.1	1.0	1.9	0.0	1.3	4.9	4.3	0.4
2	24.6	75.4	1.4	1.9	0.3	1.4	2.7	4.2	0.4
6	27.8	72.2	2.4	1.9	0.8	1.4	3.1	4.2	0.5
10	31.7	68.3	3.2	1.8	1.0	1.5	3.9	4.1	0.6

Table S9. Measured values of cumulative % drug released, % drug remaining, square root time, log cumulative % drug remaining, log time, log cumulative % drug released, % drug released, cube root of % drug remaining (W_t) and W_0-W_t parameters to determine the drug release kinetics for PEG-Lip-DOX/CB. W_0 and W_t are the initial and remaining amount of drug in the pharmaceutical dosage form at times 0 and t, respectively.

Time (hr)	cumulative % drug released	% drug remaining	Square root time	log Cumu % drug remaining	log time	log Cumu % drug released	% Drug released	Cube Root of % drug Remaining (W_t)	W_0-W_t
0	0	100	0.0	2.0	0.0	0.0	100	4.6	0.0
0.25	9.8	90.2	0.5	2.0	-0.6	1.0	9.8	4.5	0.2
0.5	17.9	82.1	0.7	1.9	-0.3	1.3	8.1	4.3	0.3
1	19.6	80.4	1.0	1.9	0.0	1.3	1.7	4.3	0.3
2	21.2	78.8	1.4	1.9	0.3	1.3	1.6	4.3	0.4
6	24.2	75.8	2.4	1.9	0.8	1.4	3	4.2	0.4
10	27.5	72.5	3.2	1.9	1.0	1.4	3.3	4.2	0.5
